# Amputation at the Hip Joint

**Published:** 1902-07-05

**Authors:** 


### AMPUTATION AT THE HIP JOINT.

Putting on one side the shock and the sudden alteration of the assimilative processes which the removal of so large a portion of the body as the whole of one lower extremity must involve, the chief danger of amputation at the hip joint lies in the chance of hæmorrhage from the great vessels which must be divided, and the chief anxiety felt by the surgeon, at any rate when he has to operate with strange assistants, lies in the fact that according to the older methods it is on the assistants that the duty of restraining this hæmorrhage mostly falls. It no doubt was a great improvement when the plan was introduced of dividing the soft parts in the middle of the thigh and enucleating the femur through a long external incision, the vessels being controlled meanwhile by the rubber band, but Mr. Edmund Owen[1] has gone a step further. According to the method he now describes, the crural sheath is first opened through an incision running vertically down from Poupart's ligament, and the common femoral vessels are secured in separate ligatures. Then a circular sweep is made round the thigh a little above the middle, straight on to the femur, the bone being sawn through at the same level, the limb being thus cut off square. An incision is then made on to the femur from the top of the great trochanter downwards, and the bone is enucleated. Some small branches of the sciatic, gluteal, or circumflex iliac arteries may require ligature, but this is a small matter; the great vessels are secured from the very beginning of the operation, and, what is in some cases a matter of great importance, they are secured by the surgeon himself. If the operation is being done for tuberculous or septic inflammation the periosteal and muscular attachments to the femur may be separated by a blunt raspatory, but if for sarcoma the surgeon would have to use the knife and keep it well wide of the bone.

[1] Lancet, June 28.

